# Perspective of HLA-G Induced Immunosuppression in SARS-CoV-2 Infection

**DOI:** 10.3389/fimmu.2021.788769

**Published:** 2021-12-06

**Authors:** Aifen Lin, Wei-Hua Yan

**Affiliations:** ^1^ Biological Resource Center, Taizhou Hospital of Zhejiang Province, Wenzhou Medical University, Linhai, China; ^2^ Key Laboratory of Minimally Invasive Techniques & Rapid Rehabilitation of Digestive System Tumor of Zhejiang Province, Taizhou Hospital of Zhejiang Province, Linhai, China; ^3^ Medical Research Center, Taizhou Hospital of Zhejiang Province, Wenzhou Medical University, Linhai, China

**Keywords:** HLA-G, immune receptor, SARS-CoV-2, COVID-19, immune suppression

## Abstract

COVID-19, the disease caused by severe acute respiratory syndrome coronavirus 2 (SARS-CoV-2), has threatened public health worldwide. Host antiviral immune responses are essential for viral clearance and disease control, however, remarkably decreased immune cell numbers and exhaustion of host cellular immune responses are commonly observed in patients with COVID-19. This is of concern as it is closely associated with disease severity and poor outcomes. Human leukocyte antigen-G (HLA-G) is a ligand for multiple immune inhibitory receptors, whose expression can be upregulated by viral infections. HLA-G/receptor signalling, such as engagement with immunoglobulin-like transcript 2 (ILT-2) or ILT-4, not only inhibit T and natural killer (NK) cell immune responses, dendritic cell (DC) maturation, and B cell antibody production. It also induces regulatory cells such as myeloid-derived suppressive cells (MDSCs), or M2 type macrophages. Moreover, HLA-G interaction with CD8 and killer inhibitory receptor (KIR) 2DL4 can provoke T cell apoptosis and NK cell senescence. In this context, HLA-G can induce profound immune suppression, which favours the escape of SARS-CoV-2 from immune attack. Although detailed knowledge on the clinical relevance of HLA-G in SARS-CoV-2 infection is limited, we herein review the immunopathological aspects of HLA-G/receptor signalling in SARS-CoV-2 infection, which could provide a better understanding of COVID-19 disease progression and identify potential immunointerventions to counteract SARS-CoV-2 infection.

## Introduction

COVID-19, the disease caused by the highly contagious virus “severe acute respiratory syndrome coronavirus 2” (SARS-CoV-2), has become a serious global public health concern (1). Although unprecedented comprehensive virus transmission prevention measures have been strictly implemented, such as travel restrictions, public social distancing, personal hygiene, and patient quarantine requirements, the virus has spread widely and caused more than 5,054,267 deaths worldwide since its outbreak in December 2019 (2–4).

The clinical manifestation of COVID-19 can be asymptomatic, mild to moderate, or severe, or critical pneumonia with symptoms of acute respiratory distress syndrome, multi-organ failure, and/or shock (5). Among patients with COVID-19, risk factors such as advanced age and pre-existing conditions are associated with increased disease severity (6). From an immunological perspective, a host’s innate and adaptive immune responses are indispensable in controlling viral infection and disease progression. However, abnormal host immune responses are common in patients with severe COVID-19, including cytokine storm resulting from hyper-inflammatory immunological humoral reactions, as well as impairment of cellular antiviral immune responses (7, 8). Our previous studies revealed that total lymphocytes, CD3+, CD4+, and CD8+ T cells were dramatically lower among patients with severe COVID-19 than among non-severe patients at admission. These cells returned to normal levels by the second week after discharge, however, lower CD8+ T cell count is an independent risk factor for longer viral positivity duration and is related to an increased risk for discharged patients with SARS-CoV-2 re-positivity (9–11). Pro-inflammatory cytokines and chemokines, such as interleukin (IL)-1β, IL-6, IL-8, tumour necrosis factor (TNF)-α, macrophage inflammatory protein (MIP) 1α/CCL3, interferon (IFN)-γ-induced protein 10 (IP10), monocyte chemoattractant protein 1 (MCP1), granulocyte colony-stimulating factor (G-CSF), and granulocyte-macrophage colony-stimulating factor (GM-CSF), were substantially increased (12, 13). Moreover, antiviral cellular immune responses were compromised because of the following: (a) the dramatically decreased absolute number of CD3+ lymphocytes, subpopulations of CD4+ and CD8+ T cells, CD3+CD56+ NKT cells, B cells, and natural killer (NK) cells (14, 15); (b) various functions were impaired and/or exhausted, such as the cytotoxicity of these immune effectors (16–18); and (c) immune regulatory cells, including myeloid-derived suppressor cells, were notably expanded in severe cases (19).

Although an increasing number of clinical and immunological findings on the immunopathological features of COVID-19 are being reported, the molecular mechanisms involved in the dysregulation of cellular immune responses against SARS-CoV-2 infection are yet to be discovered (20). During viral infection, various strategies to escape the host antiviral immune attack and to favour replication and disease progression have been developed by the virus particles (21, 22). Alteration and intervention of human leukocyte antigen (HLA) and/or its receptor expression is one of the strategies applied by viruses (23). In patients with COVID-19, HLA-E receptor CD94/NK group 2 member A (NKG2A), a member of the immune inhibitory receptors, is remarkably increased in CD8+ T and NKT cells, resulting in their functional exhaustion. Notably, high levels of NKG2A expression are significantly reduced when patients recover from the disease (18).

**Figure 1:**
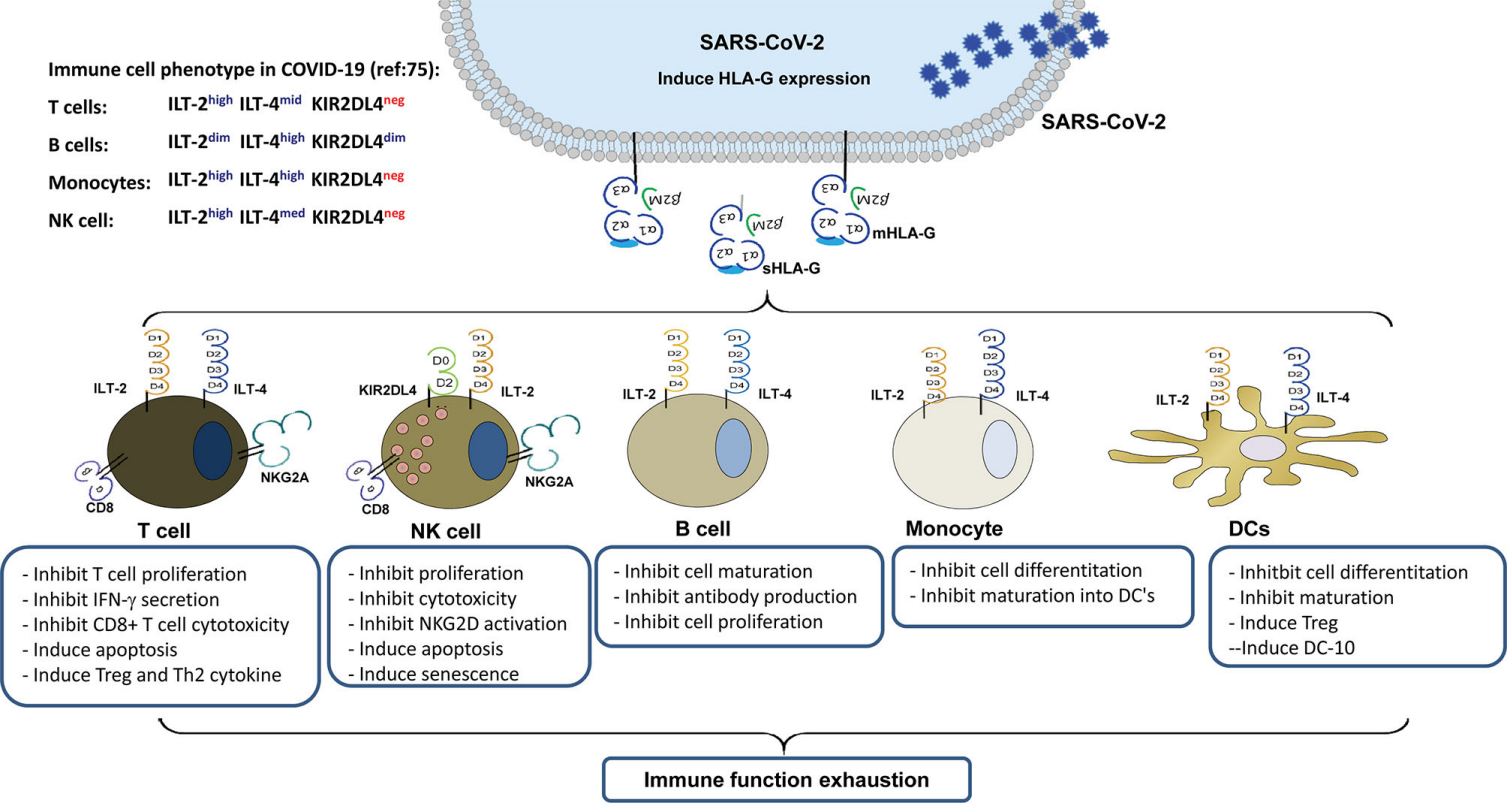
Immune suppression mediated by up-regulated HLA-G expression in virus infected cells and its interaction with immune receptors expressed on different types of immune cells during SARS-CoV-2 infection.

HLA-G is a non-classical HLA class I antigen, which is a strong immune inhibitory mediator *via* receptor signalling. Because of the alternative splicing its primary transcript, at least seven HLA-G isoforms have been identified, which include four membrane-bound (HLA-G1, HLA-G2, HLA-G3, and HLA-G4) and three soluble (HLA-G5, HLA-G6, and HLA-G7) isoforms (24). HLA-G can be upregulated by various viral infections, including SARS-CoV-2, which can render comprehensive immunosuppressive roles in favouring virus immune evasion and subsequent disease progression (25, 26). Several immune cell surface-expressed receptors have been identified that bind to HLA-G, including immunoglobulin-like transcripts-2 (ILT-2)/CD85j/LIR1, ILT-4/CD85d/LIR2, killer inhibitory receptor (KIR) 2DL4/CD158d, CD8, and CD160 (24). The glycosylphosphatidylinositol-anchored transmembrane glycoprotein receptor CD160 is closely related to the KIR2DL4, although their homology is rather weak only with 29% identity and 44% similarity (27). In this scenario, HLA-G/receptor signalling among various immune cells is important in COVID-19 pathogenesis and progression (**Figure 1**).

Herein, we focus on the implication of HLA-G/receptor signalling on immune response impairment during SARS-CoV-2 infection.

## Immune Modulation of HLA-G Molecule

The *HLA-G* gene was identified by Geraghty et al. in 1987, and HLA-G protein expression was first found in extravillous cytotrophoblasts in 1990 (28, 29). A number of studies have focused on the basic and clinical significance of HLA-G in foetal-maternal immune tolerance. Since then, immune receptors, including KIR2DL4, ILT-2, ILT-4, CD8, and CD160, have been discovered (30–34). HLA-G induced immune suppression has been extensively documented (including direct and/or indirect as well as long- and short-term), and the immunosuppressive functions of HLA-G have been well established (29, 35). The signalling between HLA-G and receptors KIR2DL4, ILT-2, ILT-4, CD8, and CD160 expressed on different types of immune cells is a fundamental prerequisite for the aforementioned immune suppressive functions of HLA-G (36). Recently, the immune inhibitory NKG2A/CD94 receptor, a well-known receptor for HLA-E, has been reported to be a new HLA-G allele-dependent receptor (37).

By directly binding to immune cell surface receptors ILT-2 or/and ILT-4, HLA-G can inhibit T cells, NK cells, and B cell proliferation, cytotoxicity, anti-inflammatory cytokines such as interferon-chemotaxis, immunoglobulin production, or MICA/NKG2D activation or cell senescence (22). HLA-G can also induce the generation of anergic and regulatory T cells (Treg), tolerogenic DCs, polarisation of M1 to M2, and Th2-type cytokine secretion (24). Moreover, HLA-G can suppress neutrophil reactive oxygen species production and the capacity for phagocytosis (38). Indirect immune suppression induced by HLA-G could be caused by multiple intracellular transfers of HLA-G from HLA-G-bearing cells to HLA-G-negative neighbouring cells or distant cells. Both allogeneic and autologous cell membranes containing HLA-G between/among cells through the process of trogocytosis have been observed, resulting in the activation of immune effector cells (T cells, NK cells, monocytes) into suppressor cells. In addition, HLA-G can be transferred by exosomes to long-distance cells, impairing the functions of immune-competent cells (39). Furthermore, indirect immune modulation induced by HLA-G could increase HLA-E expression, thus influencing the HLA-E/CD94/NKG2 receptor signalling pathway. Cell surface HLA-E expression is dependent on the binding of TAP-associated peptides derived from other HLA class I signal sequences (40). Among the different HLA class I signal peptides, the HLA-E/G-nonameric complex has a significantly higher affinity (41). However, different HLA-G isoforms may have different effects on the enhancement of HLA-E cell surface localization (42).

## Induction of HLA-G Expression in Infectious Diseases

HLA antigens are critical for both the innate and adaptive immune systems as they bind to T cell receptors to present HLA-restricted peptides to T cells and interact with NK cell receptors to modulate the functions of innate immune components, such as NK cells (43). Downregulation of the expression of the classical HLA class I antigens (HLA-A, -B, and -C) and HLA class II antigens (DP, DQ, and DR) is one of the most effective strategies for perpetuating viral infections, as it allows virus-infected cells to escape from the host immune attack led by virus-specific CD8+ T cells and blunt CD4+ T cells, which help B cells to produce virus-specific antibodies (44). The aberrant upregulation of the immune tolerant HLA-G expression is more common than the downregulation of the HLA-I and -II antigens during many infections, providing virus-infected cells a strategy to protect themselves from NK cell cytolysis (45, 46).

Mechanisms underlying the regulation of HLA-G protein expression is quite complex. HLA-G protein expression can be driven by specific *HLA-G* genetic polymorphisms, and extracellular and intracellular signals. *HLA-G* polymorphisms such as a rs66554220, a 14-bp insertion/deletion in the *HLA-G* exon 8 involved in the stability of *HLA-G* mRNA, where *HLA-G* mRNA is more stable with the 14-bp deleted allele and related to HLA-G protein expression (47–49). MicroRNAs miR-148a,miR-148b, miR-152, miR-133a, miR-628-5p, and miR-548q have been reported to regulate HLA-G expression (50). Among them, position +3142 (C>G, rs1063320) has high binding affinity to microRNAs miR-148a,miR-148b, andmiR-152, which is related to the suppression of HLA-G production (51). In addition to the microRNAs, specific RNA binding proteins, such as the heterogeneous nuclear ribonucleoprotein R (HNRNPR), which can bind *HLA-G* in 3’ untranslated regions (UTR) and stablize the HLA-G transcripts, and increase HLA-G expression (52). HLA-G promoter region includes several specific regulatory elements. HLA-G promoter heat shock element can response to heat shock proteins, and dexamethasone and progesterone can interact with a unique progesterone response element to regulate HLA-G protein expression (53, 54). Also, extracellular environmental factors such as cytokine IL-10, IFNs, indolamine 2, 3-dioxygenase (IDO), granulocyte-macrophage colony-stimulating factor (GM-CSF), hypoxia and demethylation condition affects HLA-G protein expression (48, 55–58). However, peripheral sHLA-G levels are not significantly different between male and female individuals (49, 59). Virus itself or viral gene products such as U94 viral gene of human herpesvirus 6 (HHV-6) can induce HLA-G activation by recognizing an *HLA-G* promoter consensus sequence (60). Through no specific mechanism for HLA-G up-regulation has been outlined during the SARS-CoV-2 infection, COVID-19 patients peripheral circulation highly increased cytokines such as IL-10, GM-CSF and IDO could be factors involved in the HLA-G expression modification (61, 62).

Upregulation of both virus-infected cell surface membrane-bound HLA-G and peripheral soluble HLA-G expression has been observed in various viral infectious diseases, such as human immunodeficiency virus type 1 (HIV-1), herpes simplex virus-1, rhabdovirus, human cytomegalovirus, hepatitis B and C virus, and influenza A virus (63). HLA-G expression in monocytes and T cells of HIV-1 infected individuals was much higher than that in healthy controls (64). Recent studies have shown that increased HLA-G expression on monocytes could be induced by highly active antiretroviral therapy (HAART), and that cell surface HLA-G expression is more stable than that of the other HLA molecules owing to its resistance to HIV-1 derived protein Nef degradation (65, 66). Moreover, soluble HLA-G could inhibit myeloid dendritic cell function, and higher peripheral circulating sHLA-G levels were shown to be linked to the rapid progression of HIV-1 infection (67, 68).

In the context of SARS-CoV-2 infection, there is currently limited information on the biological and clinical significance of HLA-G. In a previous study using global transcriptomic analysis, Josset et al. (26) found that SARS-CoV-2 and Middle East respiratory syndrome coronavirus (MERS-CoV) differentially activated *HLA-G* transcription in the human lung epithelial cell line Calu-3 *in vitro*. This study revealed that HLA-G transcripts could be specifically downregulated by MERS-CoV, whereas HLA-G was upregulated by SARS-CoV-2 infection. With a larger case/control cohort (2244/10220), a recent genome-wide association study (GWAS) by Pairo-Castineira et al. (69) showed that *HLA-G* (rs9380142) is a novel genetic locus, which is strongly associated with the severity of COVID-19. Association between *HLA-G* polymorphism and viral infection has been documented in a number of studies. HLA-G*01:01:08 was reported to be a risk factor for HIV-1 infection in Zimbabwean while 3’UTR 14-bp In/14-bp In was a risk factor for HIV infection in South Africans of African ancestry women (70, 71). 14-bp del/14-bp del and HLA-G*01:04:01/14-bp del genotypes, and UTR-2 and UTR-3 haplotypes were found to be the risk factors for hepatitis C virus (72, 73).

In a patient recovered from COVID-19 for four weeks, induced HLA-G expression was observed in the intestinal mucosa epithelial cells and lymphocytes at the sites corresponding to SARS-CoV-2 positivity (74). In our case report, the dynamics of HLA-G and expression of its receptors ILT-2, ILT4, and KIR2DL4 on peripheral immune cell subpopulations in a critical COVID-19 case to convalescence were analysed. Our data showed that HLA-G expression in peripheral immune cells fluctuates along with the status of the disease that the percentage of HLA-G-positive T cells, B cells and monocytes presented a high-low-high pattern, while the percentage of receptors ILT2-, ILT4- and KIR2DL4-expressing immune cells remained relatively stable (75). Moreover, sHLA-G has been found to be significantly elevated in patients with COVID-19 and is related to disease severity (76). Notably, a very recent study by Bortolotti et al. (77) reported that an increased peripheral blood sHLA-G level was associated with an improved outcome in patients with COVID-19, which might be a result of reduced neutrophil adhesion to activated endothelia by sHLA-G as well as interaction with the receptor CD160 (**Table 1**).

**Table 1:** Current available studies on HLA-G expression in patients with COVID-19.

Study method, subjects and size	Results and Implication for HLA-G expression	Reference
A 50s male patient had a positive for SARS-CoV-2 4 days after the start of symptoms. After 4-week-negative, he was admitted due to stomach pain, and a histologic examination was performed after colonoscopy.	HLA-G expression was found in intestinal mucosa epithelial cells and in some lymphocytes, in correspondence with SARS-CoV-2–positive sites. In submucosa, HLA-G expression was detectable only in few lymphocytes. Induction of HLA-G expression at the site of SARS-CoV-2 infection might be a cause of the COVID-19-dependent bleeding.	(72)
A 55-year-old female patient with critical COVID-19 admitted seven days after the onset of symptoms. Dynamics of HLA-G and its receptors ILT2, ILT4 and KIR2DL4 expression in peripheral immune cells with flow cytometry, and the outcomes of the patient during the 23-day ICU treatment.	The percentage of HLA-G+ T cells (median: 6.29%; range: 1.18-11.2%), B cells (median: 5.93%; range: 2.38-10.50%) and monocytes (median: 9.73%; range: 5.51-12.20%) is of a high (at admission)–low (during hospitalization)–high ( convalescence) pattern, while the percentage of receptors ILT2-, ILT4- and KIR2DL4-expressing cells remained more stable.	(73)
103 COVID-19 patients and 105 healthy controls were included in the case-control study.	sHLA-G were significantly increased in COVID-19 patients compared to controls (19.3 vs. 12.7 ng/mL; p <0.001). No statistical difference was observed between sHLA-G and gender, BMI, chronic disease, or ABO and Rh blood groups. Patients in the quartiles >50–75% and >75% of sHLA-G level were more likely to have COVID-19.	(74)
An investigator-initiated, prospective, single-center study. Fifty-four COVID-19 (moderate-to-severe) patients, 11 control patients that presented respiratory failure without SARS-CoV-2 infection), and 100 healthy control subjects. Serum sHLA-G were analyzed after enrollment (T1; Baseline), and every 7 ± 2 days for an additional 2 consecutive visits (T2 and T3). Correlation between sHLA-G and clinical outcomes was evaluated.	Higher sHLA-G in COVID-19 patients compared to controls with respiratory failure (165.87 vs. 49.54ng/mL; *p* < 0.01) and healthy controls ( 165.8 vs. 20.51ng/mL; *p* < 0.001) at T1. sHLA-G at T1 did not differ between COVID-19survivors and non survivors, but significantly decreased over time in non-survivors (p = 0.036 at T2; p = 0.04 at T3). In control patients, sHLA-G levels decreased in both survivors and non-survivors over time with no statistical differences. Increased severity of COVID-19 from T1 to T2 (but not T2 to T3) was associated with a significantly decreased sHLA-G (p = 0.012). Improved clinical conditions were associated with an increased sHLA-G between T1 and T2 (p = 0.01). Increased sHLA-G reduced neutrophil adhesion to the endothelial cells.	(75)

However, the clinical significance of HLA-G and its receptor expression on immune cells among patients with COVID-19 remains largely unknown.

## Implication of HLA-G/Receptor-Mediated Immune Suppression in COVID-19

The marked manifestations of immunopathology during SARS-CoV-2 infection, particularly in patients with severe COVID-19, is a salient reduction in immune-competent cells and an overregulated production of pro-inflammatory cytokines and chemokines (78). Dramatically impaired antiviral cellular immune responses and uncontrolled pro-inflammatory humoral immunity lead to SARS-CoV-2 immune evasion and collateral local or systemic tissue damage (79). In severe COVID-19 cases, cellular immune functions are not compromised by dramatically decreased CD3+ lymphocytes, CD4+, CD8+ T, CD3+CD56+ NK T, B, and NK cells, but by the impaired and/or exhausted functions of these immune cells, which is accompanied by the expansion of myeloid-derived suppressor cells (80–82).

Cytotoxic lymphocytes, such as NK cells and CD8+ T lymphocytes, can directly target virus-infected cells, and virus-specific antibody-producing B cells are essential for viral clearance and infectious disease control. In severe COVID-19 cases, not only the absolute number but also the cytotoxicity of both NK cells and CD8+ T lymphocytes are remarkably reduced. Mazzoni et al. (16) found that peripheral circulating NK cell intracellular granzyme and perforin levels were dramatically lower in patients with severe COVID-19 than in healthy controls. At the same time, there are much higher frequencies of senescent phenotype TEMRA+ CD57+ CD8+ T cells but reduced antiviral cytokine production and cytotoxicity of CD4+, CD8+ T, and NK cells in patients with COVID-19. NK cell immune function impairment could be due to the significantly increased expression of the inhibitory receptor NKG2A, as reported by Zheng et al. (18). In that study, the functions of NK and CD8+ T cells were exhausted and exhibited lower CD107a, IFN-γ, IL-2, granzyme B, and TNF-α production, which were accompanied by highly increased expression of the inhibitory receptor NKG2A on both cells during SARS-CoV-2 infection. Importantly, in most patients with COVID-19, the reduced number of NK and CD8+ T cells was restored and the initially high level of NKG2A expression was reduced during the convalescent period after antiviral therapy.

Although detailed information on the significance of HLA-G/receptor signalling in SARS-CoV-2 infection is lacking, the multifaceted immune suppression induced by HLA-G engagement with the aforementioned receptors has provided accumulating evidence that HLA-G/receptor signalling induces immune impairment and exhaustion, and cytokine release could be of critical importance in COVID-19. Previous studies have described the profound immune suppression mediated by HLA-G interaction with ILT-2/4 in a wide range of contexts (83). The interaction and signalling could inhibit the cytotoxicity of NK and CD8+ T cells, allo-proliferation of CD4+ T cells, maturation of DCs, differentiation, proliferation, and immunoglobulin (IgA, IgG, and IgM) production by B cells (84–88). In line with this, the activation of DCs and B cells was hampered in patients with severe COVID-19, as indicated by Wang et al. (89). Moreover, HLA-G/CD8 interaction could induce the apoptosis of CD8+ T cells through the Fas/FasL pathway, which may also occur in subsets of CD8+ NK T cells (90, 91). In contrast, HLA-G/ILT-2/4 engagement could also induce the generation of CD8+CD28+ or CD4+CD25+CTLA-4+ regulatory T cells (Tregs), expansion of MDSCs, tolerogenic DC-10 induced adaptive type 1 regulatory T cells, and M2 type macrophages (92–95). A study by Tomić et al. (96) revealed that the expansion of PD-L1, ILT-3, and IDO-1-expressing monocytic MDSCs was related to the accumulation of regulatory B and T cells and poor T cell immune responses in patients with severe COVID-19. Other studies have shown that CD4^+^CD25^+^CD127^low^ Treg cells were significantly increased in patients with both mild or severe COVID-19, regardless of recovery, and that the proportion of IL-10 producing Treg was significantly increased in patients with severe COVID-19 (97, 98). However, the expression status of HLA-G and its receptors on these immune cells remains to be investigated.

Moreover, HLA-G expression in swine endothelial cells can protect them from human macrophage-mediated cytotoxicity (99, 100). Based on our preliminary study on HLA-G receptor expression in circulating immune subpopulations in a critical patient with COVID-19, the data showed that T cells can be phenotyped as ILT-2^high^ILT-4 ^mid^KIR2DL4^dim^, B cells as ILT-2 ^mid^ILT-4^high^KIR2DL4^dim^, and monocytes as ILT-2^high^ILT-4^high^KIR2DL4^dim^. However, the cell surface expression levels of these receptors remained relatively stable from the critical stage to convalescent stage and irrespective of the viral load in SARS-COV-2 infection (75). The marginal KIR2DL4 expression observed in T cells, B cells, and monocytes in our study is in agreement with previous reports and is mainly located intracellularly but detectable upon IL-2 activation of NK cells (101). The activation signal resulting from HLA-G/KIR2DL4 interaction not only initiates the production of robust pro-inflammatory cytokines and chemokines, such as IFN-γ, TNF-α, IL-1β, IL-6, IL-8, MIP-3α, MIP-1δ, MIP-1α, and MIP-2β, but also leads to cell senescence and cell cycle arrest in NK cells (102, 103).

HLA-G allelic products also affect the interaction with its receptors, caused by different amino acid residues, and consequently alter its biological functions. Celik et al. (104) indicated that a single amino acid difference in the α two domains of HLA-G could affect the lysis of target cells by NK cells. The data showed that a much stronger immune suppressive function was observed for the HLA-G*01:04 allele than for the HLA-G*01:01 and HLA-G*01:03 alleles. It seems reasonable with a recent finding that the binding of the HLA-G*01:04 product to NKG2A/CD94 has a higher affinity than that of HLA-G*01:01 and HLA-G*01:03 products (37). As NKG2A expression is highly associated with the severity of COVID-19, these findings indicate that the genetic variation of HLA-G could be linked to susceptibility to disease and host immune response regulation during SARS-CoV-2 infection (105).

## Conclusions

Since the outbreak of the worldwide COVID-19 pandemic in December 2019, it has claimed more than 5,054,267 lives (4). More insights into the ever-increasing clinical characteristics and laboratory findings on COVID-19 are being reported, which show that immune-competent cell function impairment and/or exhaustion is one of the major features of COVID-19 pathogenesis (7). However, the mechanisms underlying immunological abnormalities remain largely unknown. As reported in previous studies, viruses have developed effective strategies to hide from host antiviral immune responses and survive during infection (106). One strategy successfully deployed by viruses for immune evasion is the impairment of the classical HLA class I and II antigens to hide infected cells from T cell recognition, and the induction of non-classical HLA class I antigen HLA-G, a ligand for immune inhibitory receptors differentially expressed on almost all subsets of immune cells. Consequently, differential alteration in HLA antigen expression by viral infection makes the host antiviral immune system vulnerable (22, 107).

Synergistic suppression effects induced by HLA-G/receptor signalling are well recognised. These effects include the inhibition of cell proliferation and differentiation and the induction of cell apoptosis and senescence, which could be involved in significant decrease or even exhaustion of immune-competent cells such as T cells, NK cells, B cells, and macrophages in patients with COVID-19. Other effects include the inhibition of T and NK cell cytotoxicity, antibody production by B cells, and induction of regulatory cells and expansion of MDSCs, which might be linked to the functional impairment of effector cells, such as T, NK, and B cells, during SARS-CoV-2 infection. However, more information on HLA-G and its receptor status is necessary for future clinical investigations and basic science studies.

## Perspectives

**Figure 2:**
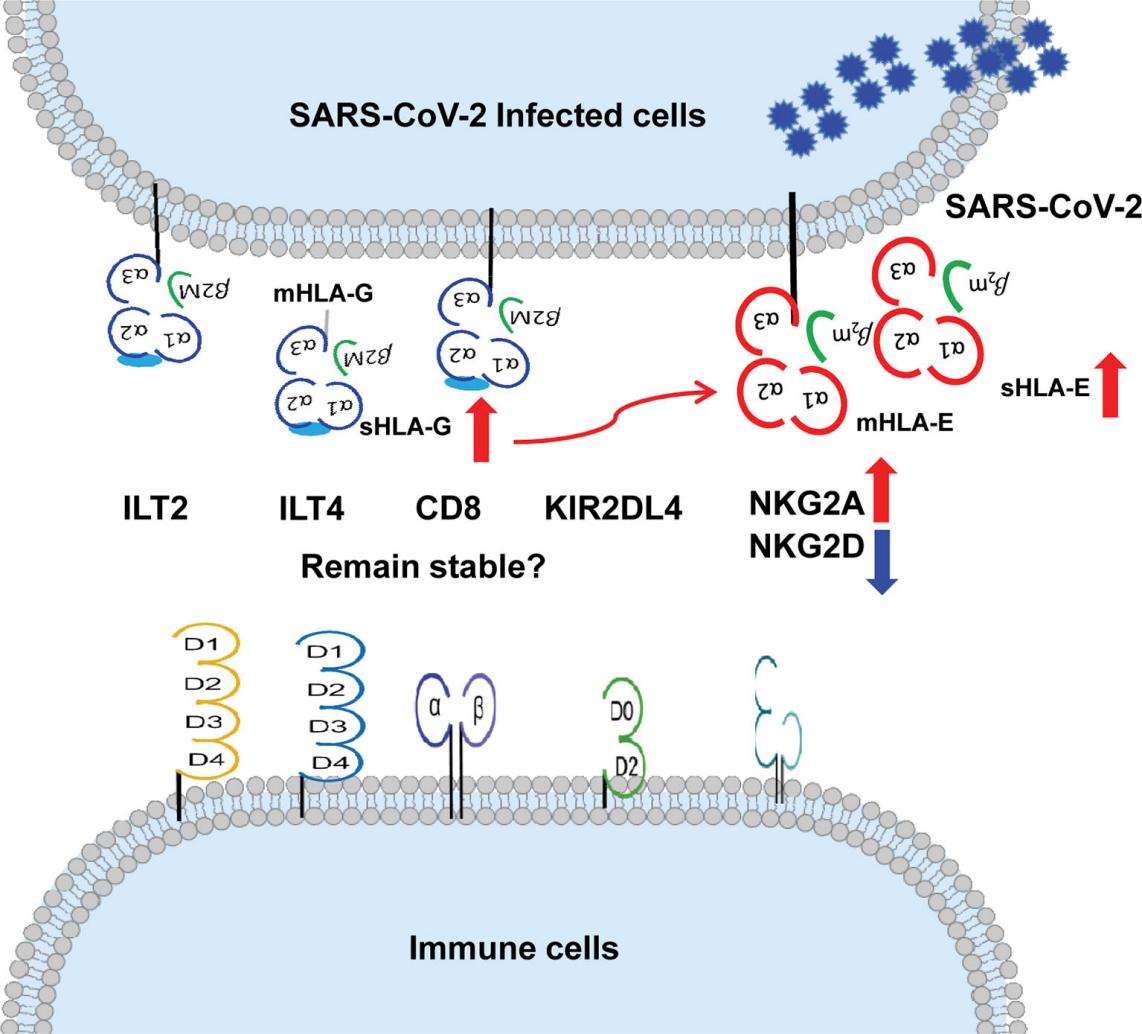
More evidence can be expected on the relationship between up-regulation of HLA-G and HLA-E expression and their immune receptors on immune cells during SARS-CoV-2 infection. ↑up-regulation. ↓down-regulation.

More evidence can be accumulated to solidify the basic and clinical aspects of HLA-G in COVID-19 progression and outcome. Aspects expected to be explored include (a) HLA-G expression is reported to be correlated with the progression of various infectious diseases (108, 109). We hypothesise that cell surface HLA-G and circulating soluble HLA-G levels are related to the severity, outcome, or viral load in patients with COVID-19. (b) The upregulation of HLA-G expression by cytokines, such as IFN-γ, IL-6, and IL-10, is dramatically increased in patients with severe COVID-19 (110, 111). We hypothesise that HLA-G expression is related to cytokines in patients with COVID-19. (c) Recently identified HLA-G allelic product-dependent receptor NKG2A has been observed to be dramatically increased in patients with COVID-19 (18). What is the status of other HLA-G receptors, such as ILT-2, ILT-4, and KIR2DL4, and their relationship with disease progression? and (d) Given HLA-E-CD94/NKG2A axis plays critical roles in COVID-19 and HLA-E cell surface expression depends other leader sequence peptides, particularly derived from HLA-G (112), what is the relationship between HLA-G and HLA-E expression? **(** **Figure 2** **)**.

In this context, the clinical trial “HLA-G Immuno-Inhibitor Checkpoint Study in Patients With COVID-19 Infection: Molecular and Cellular Assessment (HLA-G-COVID) (NCT04613297)” has been started to evaluate the clinical significance of HLA-G and receptors ILT-2 expression on CD4^+^ and CD8^+^ lymphocytes, and the levels of peripheral sHLA-G and plasma HLA-G-bearing microvesicles among COVID-19 uninfected patients, non-hospitalized COVID-19 infected patients and hospitalized COVID-19 infected patients. With this clinical trial, further understanding of the significance of HLA-G and its receptors in COVID-19 patients can be expected.

The impaired immune functions of NK and T cells resulting from ILTs and NKG2A expression have been reported to be involved in virus immune evasion and related infectious disease progression (113–116). Fortunately, ILTs, NKG2A, and HLA-G targeted immunotherapy and signalling pathway blockades are already in development in clinical trials for cancer immunotherapy. In previous preclinical investigations, blocking tumour cell-expressed HLA-G or immune cell surface ILT2/4 with specific antibodies could restore the functions of NK cells or T cells against target cells (36). Furthermore, blocking NKG2A with monalizumab, an anti-NKG2A monoclonal blocking antibody, can significantly restore the cytotoxic function of NKG2A+ NK and T cells (117–119). Along with these findings, the application of ILTs and NKG2A targeted blocking antibodies could be an additional intervention to mitigate the severity of COVID-19. Finally, a clinical phase I trial with an HLA-G blockade antibody, TTX-80, was launched in July 2020 for patients with advanced solid cancer (120), which shed new light on the restoration of exhausted immune responses induced by HLA-G in diseases such as cancers or viral infections.

We hope that our review will provide a much better understanding of the immune pathogenesis of COVID-19, and thereby help in the development of immunointerventions to counteract SARS-CoV-2 infection.

## Author Contributions

W-HY and AL conceived and designed the review. W-HY made the figures. AL and W-HY drafted and revised the manuscript and approved it for publication. All authors contributed to the article and approved the submitted version.

## Funding

This work was supported by grants from the Science and Technology Bureau of Taizhou (1901ky01; 1901ky04, 1901ky05).

## Conflict of Interest

The authors declare that the research was conducted in the absence of any commercial or financial relationships that could be construed as a potential conflict of interest.

## Publisher’s Note

All claims expressed in this article are solely those of the authors and do not necessarily represent those of their affiliated organizations, or those of the publisher, the editors and the reviewers. Any product that may be evaluated in this article, or claim that may be made by its manufacturer, is not guaranteed or endorsed by the publisher.
